# Antiretroviral Therapy for Refugees and Internally Displaced Persons: A Call for Equity

**DOI:** 10.1371/journal.pmed.1001643

**Published:** 2014-06-10

**Authors:** Joshua B Mendelsohn, Paul Spiegel, Marian Schilperoord, Nadine Cornier, David A. Ross

**Affiliations:** 1 MRC Tropical Epidemiology Group, Department of Infectious Disease Epidemiology, London School of Hygiene & Tropical Medicine, London, United Kingdom; 2 Public Health and HIV Unit, United Nations High Commissioner for Refugees, Geneva, Switzerland

## Abstract

Joshua Mendelsohn and colleagues discuss the moral, legal, and public health principles and recent evidence that strongly suggest that refugees and internally displaced people should have equal access to HIV treatment and support as host nationals and give detailed recommendations for refugees and internally displaced people accessing antiretroviral therapy in stable settings.

*Please see later in the article for the Editors' Summary*

**Summary Points**

- Available evidence suggests that refugees and internally displaced persons (IDPs) in stable settings can sustain high levels of adherence and viral suppression.
- Moral, legal, and public health principles and recent evidence strongly suggest that refugees and IDPs should have equitable access to HIV treatment and support.
- Exclusion of refugees and IDPs from HIV National Strategic Plans suggests that they may not be included in future national funding proposals to major donors.
- Levels of viral suppression among refugees and nationals documented in a stable refugee camp suggest that some settings require more intensive support for all population groups.
- Detailed recommendations are provided for refugees and IDPs accessing antiretroviral therapy in stable settings.

## Background

For people living with HIV and AIDS (PLHIV), treatment with antiretroviral therapy (ART) can result in viral suppression, prolonged life expectancy, and reduced HIV transmission [1]–[3]. To realize these benefits, PLHIV require regular access to medications and supportive services. Within conflict-affected settings, there are unique challenges to providing, accessing, and adhering to ART [4],[5]. An estimated 1.5 billion people live in countries impacted by violent conflict and 45.2 million are forcibly displaced as a result of persecution, conflict, violence, and/or human rights violations [6],[7]. In 2006, 1.8 million PLHIV were affected by conflict, disaster, or displacement. Half of countries (7/15) with the largest number of PLHIV were affected by a major conflict between 2002 and 2006 [8]. Conflict-affected persons reside in areas of recent or active conflict, or in a post-conflict camp, urban, or rural setting, and may be accorded an official status depending on their situation [9],[10]. Refugee status is available to individuals with “a well-founded fear of being persecuted for reasons of race, religion, nationality, membership of a particular social group or political opinion, is outside the country of his/her nationality and is unable or unwilling (owing to such fear) to avail him/herself of the protection of that country” [11]. Internally displaced persons (IDPs) are citizens who have “been forced or obliged to flee or to leave their homes or places of habitual residence, in particular as a result of or in order to avoid the effects of armed conflict, situations of generalized violence, violations of human rights or natural or human-made disasters, and who have not crossed an internationally recognized State border” [12]. A protracted refugee situation occurs when >25,000 refugees of the same nationality have been in exile for ≥5 years. At the end of 2012, 6.4 million refugees, or 61% of the global total of 10.5 million, lived in a protracted situation [7]. In the post-emergency phase of forced displacement, refugees and IDPs live in relatively “stable” settings, meaning that exceptional measures are no longer required to remove threats to life or well-being [13]. Here, we synthesize norms of practice with evidence on the impact of ART programs in stable settings, including our own experiences delivering and evaluating ART as part of the United Nations High Commissioner for Refugees (UNHCR) mandate. We then propose operational recommendations for improving HIV treatment outcomes among refugees and IDPs in stable settings. Moral, legal, and public health principles, in combination with recent evidence, provide a clear rationale for sustainable treatment provision and adequate support for refugees and IDPs.

## Equity and Sustainability

Debates over the merits of providing ART to refugees and IDPs have echoed earlier discussions on treatment scale-up in resource-constrained settings [14]. Four main arguments support the provision of ART to these groups: (1) the evidence for clinical benefit, reduced transmission, and cost-effectiveness supports the scaling-up of treatment to all in need [15],[16]; (2) where scarcity exists, principles of fairness should be applied uniformly to allocation of treatment [17],[18]; (3) the right to health, including the principle of access to essential medicines, clearly articulates a rationale for providing access to life-saving interventions for PLHIV [19]–[21]; and (4) the Convention Relating to the Status of Refugees enshrines a principle of equity whereby hosting countries should provide refugees with a similar standard of medical care to the standard that is routinely available to host nationals [11],[12].

In practice, however, countries do not uniformly provide access to ART within their borders [22], and international assistance can be slow in reaching these groups. Compared with unaffected countries, conflict-affected countries received less than half of the funds provided through overseas development assistance (ODA) for reproductive health services in the period 2006–2008 [23]. Funding cycles are also temporary. Funding provided for refugees and local populations by the World Bank through the Great Lakes Initiative on AIDS for refugees and surrounding host populations recently ended, leading to a resource gap. Given budget pressures, national governments may give preferential treatment to nationals and/or non-minorities. Funds provided by the major donors such as Global Fund to Fight AIDS, Tuberculosis and Malaria (Global Fund), and the President's Emergency Plan for AIDS Relief (PEPFAR) are less likely to find groups who are excluded from national funding proposals, an omission that is more likely to occur when such marginalized groups are also excluded from HIV National Strategic Plans (NSPs). Of NSPs issued in the ten year period from 1998 to 2008 by African countries, only 52% and 43% mentioned refugees and IDPs, respectively [24],[25].

## Evidence for Action

Evidence of effectiveness is needed to support arguments for scaling-up treatment and support. Findings from studies conducted among refugees and IDPs showed that 87%–99.5% of clients studied had achieved ≥95% adherence and positive treatment outcomes, as measured by survival and CD4 gains [26]. However, outside of high-income settings, very few published studies have used virologic outcomes to assess program performance. One study that compared citizens with “foreigners” who relocated to Johannesburg found higher levels of viral suppression among the displaced group (76% versus 58%, *p* = 0.02) [27].

Our direct experience comes from delivering and evaluating ART programs as part of UNHCR's mandate to protect refugees. We conducted two evaluations using virologic outcomes in two stable refugee settings (Table 1). In a stable urban setting (Kuala Lumpur, Malaysia), similar proportions of refugees and host nationals accessing treatment from a shared clinic achieved viral suppression (83% overall), while proportions adhering to treatment were similar according to pharmacy refill records and self-reports [28]. In a stable refugee camp (Kakuma, Kenya), considerably lower proportions (50% overall) of both groups on treatment for ≥25 weeks were virologically suppressed [29]. This discrepancy may have been due to unverified adherence lapses, background levels of drug resistance, or high ambient medication storage temperatures [29]. These evaluations, in combination with evidence from other settings, suggest that treatment outcomes among stable refugees or IDPs, and host nationals, are similar when treatment and support is accessed from a shared clinic. Therefore, similar levels of viral suppression should be expected, although more challenging settings will require more intensive and specialized support for all population groups. Even in stable settings, disruptions may still occur. During the 2008 post-election violence in Kenya, an otherwise stable setting, people on ART appeared to have difficulties locating and/or taking their treatment. During this period of instability, 16% of clients on ART interrupted their treatment, as compared with 10% during a stable comparison period, and mortality rates increased [30],[31]. In stable settings at increased risk for disruption, the potential negative effects of treatment interruptions may be lessened if the period of disruption is short, there are strong contingency plans in place for ensuring continuing access to treatment, and people on ART have been educated on how to best manage their treatment in these challenging circumstances.

**Table 1:** Results of UNHCR-sponsored evaluations conducted in Malaysia and Kenya.

Outcome	Malaysia			Kenya		
	Refugee	Host National	*p*-Value	Refugee	Host National	*p*-Value
Viral suppression^a^	81% (98/121)	84% (105/125)	0.54	58% (34/59)	43% (31/72)	0.10
≥95% pharmacy refill^b^ (24 months)	74% (101/136)	66% (95/143)	0.15	85% (62/73)	74% (64/86)	0.09
≥95% self-reported adherence^b^ (past month)	72% (110/153)	70% (104/148)	0.79	62% (45/73)	28% (24/86)	0.002

a ≥25 weeks on treatment; cut-offs: Malaysia, 40 copies/ml and Kenya, 5,000 copies/ml. The difference in cut-offs was due to collection method: blood plasma was collected using routine phlebotomy services in Malaysia and whole blood was collected as dried blood spots in Kenya. Note that the 5000 copies/ml cut-off used here differs from the 1000 copies/ml reported previously [29]. A higher cut-off has been used to conform to current guidelines [36].

b ≥30 days on treatment.

## Recommendations

Despite the challenges, there is now general agreement on the public health, humanitarian, and human rights arguments that support access to ART for refugees and IDPs when clinically indicated [32]–[35]. To this end, donors, health care workers, and hosting countries should collaborate on strategies for expanding access and providing the necessary supportive services. On the basis of our experiences implementing ART interventions for refugees and IDPs in stable post-conflict settings, our own rigorous evaluations of two such programs, and the experiences of others in this area, we have proposed a set of operational recommendations in support of ART programs for refugees and IDPs situated in stable settings (Table 2). These recommendations are intended to complement WHO's new consolidated guidelines on the use of antiretroviral drugs [36].

**Table 2:** Recommendations for provision of antiretroviral therapy to refugees and IDPs in stable settings.

Theme	Recommendation	Stakeholder(s)
**Initiation and distribution of ART**	1. Start or continue treatment as soon as it is clinically indicated. Scale-up HIV counseling and testing to facilitate treatment initiation according to national guidelines. Ensure equitable access to treatment and testing for refugees and IDPs, and key populations among them (e.g., gay men, men who have sex with men, people who inject drugs, transgender persons, and sex workers). Absence of routine laboratory monitoring should not be used as a reason to deny treatment [55].	**Health care providers; MOH; donors**
	2. Distribute ART through partnerships with decentralized pharmacy networks to reduce travel burden. Where multi-tablet regimens are indicated, facilitate refills of full prescriptions at one pharmacy location.	
	3. Deliver medication directly to clients who are disabled, or who do not regularly attend the clinic because of stigma, disability, or prohibitive cost.	
	4. Disburse medications with clear and validated pictorial dosing instructions that may be understood by individuals from different linguistic and educational backgrounds.	
	5. Carefully manage medication storage conditions to ensure that appropriate temperatures are maintained. Decentralize storage facilities where there is a risk of local insecurity (e.g., conflict, theft).	
**Adherence support and monitoring**	6. Support point-of-care patient laboratory monitoring for both pre-ART and ART clients. Use viral load testing to diagnose or confirm treatment failures [36].	
	7. Update clinical guidance for treatment continuation given length of possible treatment interruption and client treatment history.	
	8. Adopt a routine adherence monitoring system using a counselor-administered or self-administered visual analogue instrument and pharmacy refill measures. The system should be rights-based (e.g., voluntary consent required for participation). Integrate medical and pharmacy records to facilitate identification of missed prescription refills. If multiple pharmacies are accessed by individual clients, link the pharmacy records to facilitate accurate monitoring of adherence to refill schedules.	
	9. Train, support, and evaluate community health and counseling teams. Sensitize counselors to local gender, ethnic, and linguistic challenges. Work in teams to ensure continuity of counseling in the event of sudden leave or dislocation. Avoid parallel programs.	**Health care providers; pharmacies; MOH; donors**
	10. Develop a basic package of locally appropriate adherence interventions, and a more intensive package for groups at high risk of adherence lapses, focusing on those who live far from clinics. Pilot-test counseling, mobile phone SMS, and peer-support interventions to improve adherence. Peer-support groups for both clients and health care providers can facilitate troubleshooting of local challenges.	
	11. Provide training to ART clients in identification of non-reputable treatments and best practices for managing treatment interruptions. Provide an emergency toll-free hotline for clients and treatment providers. Provide clients with a travel kit that contains: (i) a “Health Passport” that details ART history and laboratory tests; (ii) a regional clinic roadmap with details of alternative local and cross-border clinics where ART is available; (iii) a transfer-out or referral letter; (iv) a three month buffer supply of the current ART regimen; (v) a dual nucleoside regimen for “covering the tail” of non-nucleoside reverse transcriptase inhibitor based regimens in case of treatment interruption; (vi) a tracking form with a self-addressed, stamped envelope to be submitted upon returning to the “home” clinic or to be posted to the home clinic upon enrolment in a new program [56]. Set up referral systems for those who are repatriated, resettled, or returning home within the same country.	
**Law enforcement**	12. Provide interventions within law enforcement institutions of the rights accorded to refugees, IDPs, and asylum-seekers.	**MOH; law enforcement agencies; United Nations agencies**
**Health systems**	13. Plan for changes in treatment guidelines that may affect ART supply chains. Allow a transitional overlap between the discontinuation of an old regimen and the implementation of a new regimen. Avoid situations where stock-outs or the threat of stock-outs encourage forced alteration of refill schedules.	**MOH; health care providers; pharmacies; donors**
	14. Include diagnosis and treatment of tuberculosis as an integrated one-stop service along with HIV services, with particular attention to infection control.	
**Funding**	15. Distinguish between different conflict-affected persons and forcibly displaced groups in all proposals to major donors, indicating specific activities for each group. Formalize responsibilities and identify key stakeholders within national governments, non-governmental organizations, and United Nations agencies.	**MOH; United Nations agencies**
	16. Develop regional initiatives to fund linkages between clinics and pharmacies. Share national and international resources so refugees/IDPs and local host communities may collectively benefit from interventions.	
**Research**	17. Build on the evidence-base with prospective cohorts, intervention studies, studies among children and young people, studies among key populations, and studies of drug-resistance.	

ART, antiretroviral therapy; MOH, Ministry of Health; NSP, National Strategic Plan; PEPFAR.

First, treatment should be offered to all refugees and IDPs who meet national guidelines (Recommendation 1). Despite the known effectiveness of ART, some governments may be reluctant to provide it to these groups owing to a misplaced belief that starting or continuing them on treatment may make it difficult for them to return home and that ART may serve as a pull factor, drawing additional refugee claimants to the country. However, the majority of hosting countries (88%) provide equitable access to ART for refugees and IDPs, UNHCR has not noted such a pull factor [37], and international humanitarian law entitles refugees to equitable access. IDPs should clearly benefit from inclusion in their national HIV programs. Scaling-up of HIV counseling and testing so all who are eligible are given a chance to initiate ART at the optimal time will save lives and reduce costs by increasing survival and reducing HIV transmission [2],[3],[38]. Appropriate measures must be taken to ensure that key populations (e.g., men who have sex with men, people who inject drugs, transgender persons, and sex workers) within refugee, IDP, and host national groups also receive equitable access to treatment and testing.

ART should be available in convenient locations through decentralized networks of clinics and pharmacies (Recommendation 2) or delivered directly to clients who are unable to access the medications themselves (Recommendation 3). Dosing instructions that are designed and proven to be universally understood should be provided with each medication refill (Recommendation 4). In areas with extreme ambient temperatures, drug stocks should be stored carefully and within the temperature ranges suggested by manufacturers (Recommendation 5).

Adherence monitoring and support is crucial for achieving consistent treatment outcomes and can help to avoid costly treatment failures. Viral load testing should be used to diagnose or confirm treatment failures (Recommendation 6) and may be done on an annual basis or as often as resources will permit. Pharmacy-based and self-reported measures should also be used to routinely monitor adherence (Recommendation 8). Although adherence self-reports may overestimate adherence, they have diagnostic value, are easy to implement in clinical settings, and can help to complement pharmacy refill measures when the period between refills is lengthy or if the pharmacy data is inconsistently recorded. Both measures are consistent with previous recommendations and are associated with virologic outcomes [39]–[41].

Community health and counseling teams who are sensitized to gender, ethnic, and linguistic challenges should be mobilized (Recommendation 9). Interventions that employ mobile phone text messaging, enhanced counseling and peer-support should be pilot-tested among refugees and IDPs to assess acceptability and effectiveness [42]–[44]. Addressing travel distances to clinics and transportation costs are crucial as longer travel times and higher costs may increase the likelihood of treatment interruptions [45]–[47]. Peer-support groups for both clients and health care providers can facilitate troubleshooting of local challenges (Recommendation 10). Refugees and IDPs may travel to re-unite with family in host countries, repatriate to their home country, or resettle to a new country. Even in stable settings, proper preparations for the possibility of onward displacement are important to include in programming and will serve the dual purpose of preparing people on treatment for sudden, unexpected instability (Recommendation 11). [48] As barriers to accessing treatment and supportive services sometimes include fear of detention by law enforcement officials during routine transit in urban and rural settings, interventions are needed to increase awareness among law enforcement officials of the rights accorded to refugees, asylum-seekers, and IDPs (Recommendation 12) [49].

As treatment guidelines change, new regimens will need to be stocked and distributed. Measures should be taken to avoid situations where stock-outs or the threat of stock-outs lead to forced alteration of ART refill schedules. Remote pharmacies and clinics (e.g., refugee camps) are advised to allow a transitional overlap of between the discontinuation of an old regimen and the implementation of a new regimen (Recommendation 13). There is a lack of integration, in general, between HIV and other programs including TB services; clinical settings serving refugees and IDPs are no different [50]. Diagnosis and treatment of tuberculosis should therefore be integrated with HIV services (Recommendation 14).

Host countries are advised to include specific results-oriented interventions for refugees and IDPs in their NSPs, Global Fund, and PEPFAR proposals (Recommendation 15). These proposals should identify a plan for equitable access to ART and which agencies will be responsible for program delivery [51]. Regional initiatives can facilitate partnerships in treatment management by providing a framework for assisting those who move within a country or across an international border, while helping to monitor and distinguish clients who are successfully re-integrated into treatment programs elsewhere from those who have been lost to follow-up. By engaging in partnerships with humanitarian organizations, governments can leverage international funds for the benefit of their own citizens as well as the groups they are hosting (Recommendation 16) [52]. Finally, there is a continuing need for additional evidence. As the majority of research has been conducted among adult refugees and IDPs, there is a need for research among children and young people [53],[54]. As past studies among refugees and IDPs have largely focused on short-term outcomes, prospective cohort studies or intervention studies with virologic endpoints are needed. We are not aware of any data on rates of acquired or transmitted drug resistance among these groups (Recommendation 17).

Overall, this set of recommendations will face a range of implementation barriers including resource constraints and coordination challenges among stakeholders. However, these improvements will be necessary if sustainable treatment outcomes are to be achieved among refugees and IDPs who live in stable settings.

## Conclusions

Given recent evidence and the moral, legal, and public health arguments, refugees and IDPs situated in stable settings should have equitable access to HIV treatment and supportive services. Despite encouraging treatment outcomes among these groups across a range of settings, considerable challenges persist. Programs that are not achieving high levels of success should serve as a call for intervention, not exclusion. Few would rationally argue that challenges to providing life-saving treatment should be addressed by denying access. Since antiretroviral therapy can help prevent onward transmission of HIV to sexual partners [2], it is in the enlightened self-interest of governments that host refugees and IDPs to support programs that serve all populations within their borders to the highest possible standard.

## Abbreviations

ART: antiretroviral therapy
IDP: internally displaced person
NSP: National Strategic Plan
PLHIV: people living with HIV and AIDS
UNHCR: United Nations High Commissioner for Refugees
